# Thermal analysis of laser irradiation-gold nanorod combinations at 808 nm, 940 nm, 975 nm and 1064 nm wavelengths in breast cancer model

**DOI:** 10.1080/02656736.2021.1956601

**Published:** 2021-07-27

**Authors:** Leonardo Bianchi, Rachael Mooney, Yvonne R. Cornejo, Emiliano Schena, Jacob M. Berlin, Karen S. Aboody, Paola Saccomandi

**Affiliations:** a Department of Mechanical Engineering, Politecnico di Milano, Milan, Italy; b Department of Developmental and Stem Cell Biology, Beckman Research Institute at City of Hope, Duarte, CA, USA; c School of Engineering, Università Campus Bio-medico di Roma, Rome, Italy; d Department of Molecular Medicine, Beckman Research Institute at City of Hope, Duarte, CA, USA

**Keywords:** Laser, noninvasive thermometry, gold nanorods, *in vivo* trials, laser-tissue interaction

## Abstract

**Background:**

Photothermal therapy is currently under the spotlight to improve the efficacy of minimally invasive thermal treatment of solid tumors. The interplay of several factors including the radiation wavelengths and the nanoparticle characteristics underlie the thermal outcome. However, a quantitative thermal analysis in *in vivo* models embedding nanoparticles and under different near-infrared (NIR) wavelengths is missing.

**Purpose:**

We evaluate the thermal effects induced by different combinations of NIR laser wavelengths and gold nanorods (GNRs) in breast cancer tumor models in mice.

**Materials and methods:**

Four laser wavelengths within the therapeutic window, i.e., 808, 940, 975, and 1064 nm were employed, and corresponding GNRs were intratumorally injected. The tissue thermal response was evaluated in terms of temperature profile and time constants, considering the step response of a first-order system as a model.

**Results:**

The 808 nm and 1064 nm lasers experienced the highest temperature enhancements (>24%) in presence of GNRs compared to controls; conversely, 975 nm and 940 nm lasers showed high temperatures in controls due to significant tissue absorption and the lowest temperature difference with and without GNRs (temperature enhancement <10%). The presence of GNRs resulted in small time constants, thus quicker laser-induced thermal response (from 67 s to 33 s at 808 nm).

**Conclusions:**

The thermal responses of different GNR-laser wavelength combinations quantitatively validate the widespread usage of 808 nm laser for nanoparticle-assisted photothermal procedures. Moreover, our results provide insights on other usable wavelengths, toward the identification of an effective photothermal treatment strategy for the removal of focal malignancies.

## Introduction

Minimally invasive thermal ablation is a high-potential therapeutic procedure for cancer treatment. In this technique, a localized tumor temperature increase is provided to induce a circumscribed irreversible cell injury and, consequently, apoptosis and coagulative necrosis [1]. The optimized and controlled execution of these hyperthermic oncology treatments could show multiple benefits over conventional surgical intervention, e.g., the limitation of the operative trauma, the decrease of pain, and the minimization of fibrotic scarring tissue formation [2,3]. Moreover, the reduced invasiveness permits to circumvent the risk of surgical site infection and related complications [4]. Among the different therapeutic thermal techniques, laser ablation (LA) represents a promising therapy for the management of tumorous tissues. Here, the heat generation, based on the interaction between the specific wavelength light and the tissue, depends on different factors. The absorption capability of the tissue itself plays an important role since each tissue constituent exhibits a wavelength-dependent absorption [2,5]. For instance, wavelengths strongly absorbed by tissue are typically used for superficial treatments, whereas high optical penetration depths are required when treating deeper solid tumors [6]. The predominant chromophores in vascularized tissue (water, blood, melanin, fat, yellow pigments) present a decreased absorption coefficient in the near-infrared (NIR) region, between 800 and 1200 nm. This range of wavelengths is called ‘therapeutic window’. The advantage of operating in this part of the spectrum, characterized by reduced scattering and low absorption coefficients is the deeper penetration of light inside the tissue (e.g., in the NIR-UV range, the absorption coefficient of biological tissue ranges 0.1–10,000 cm^−1^, corresponding to a higher penetration depth for the wavelengths comprised in the NIR spectrum [7,8]).

Despite encouraging results in solid tumor treatments [6,9], the widespread usage of LA in clinical practice is limited by the necessity of enhancing the procedure selectivity. That consists of localizing the temperature increase within tumor margins while sparing the surrounding off-target healthy tissue [10]. In this concern, plasmonic photothermal therapy (PTT) is being developed, relying on the assistance of nanotechnology approaches to laser therapy, to enable tumor-specific heating. Nanostructures can be positioned in the cancer site to enhance the light-absorbance capability of that specific area. Indeed, advantageous characteristics of materials, such as gold, arise when scaling them down to the nanoscale [11], thanks to the variation of their physicochemical properties [4].

For this purpose, gold nanorods (GNRs) have shown interesting peculiarities when irradiated with NIR light [12–14]. GNRs are easily tunable and they can be synthesized with different aspect ratios (ARs), i.e., the length divided by the width of the GNR [15]. This permits the wavelength-selective light absorption in the NIR region. The use of agents that are active in this specific part of the radiation spectrum is advantageous in order to minimize the light extinction operated by intrinsic biological chromophores [16].

In GNRs-mediated photothermal ablation, different factors can affect the result of the therapy and thus need to be investigated. It is important to correlate and examine the characteristics of the therapeutic laser beam according to the shapes and dimensions of nanostructures and to evaluate the tissue thermal response in the presence and absence of nanoparticles.

On account of the therapeutic window, lasers of 800–980 nm and 1064 nm wavelengths represent the most utilized NIR radiations for tissue thermal ablation [17–19]. Whereas the 1064 nm is one of the most commonly used wavelengths for only-tissue treatments, in nanoparticle-assisted PTT studies 808 nm represents a diffusely adopted wavelength [9,20,21]. Moreover, the potential usefulness of other diode lasers, characterized by a lower penetration depth, such as 940 nm and 975–980 nm, is being investigated both for soft tissue thermotherapies [22–24] and GNR-assisted PTT [25,26]. Hence, several preclinical trials have been conducted to study the feasibility of GNRs-mediated phototherapy [10,12,20,21] and demonstrated the potential of this technique. However, a quantitative assessment of the thermal response induced in biological tissues by different combinations of laser wavelengths and GNR sizes, at the same setting parameters, could provide further insight into the development of an efficient photoablative strategy. Indeed, most of the studies in this field focus on the evaluation of the effectiveness of a specific nanoparticle-laser wavelength combination [27], employing a single laser emitter, or investigate the effect of laser-tissue interaction at different wavelengths only on *ex vivo* tissue or *in vitro* conditions [22,28]. In this way, important factors typical of the *in vivo* tissue characteristics are not adequately considered. Thus, the need to examine the phenomenon with models more representative of the real physiological conditions.

**Figure 1.**
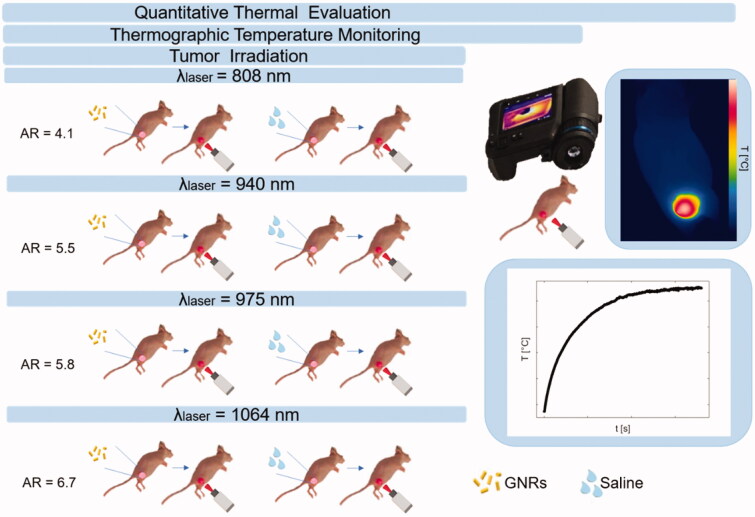
Study plan describing the experimental strategy adopted in the present work. Breast cancer tumors were grown on mice flanks and underwent injection of either gold nanorods or saline solution. The aspect ratios of the gold nanorods were selected according to the radiation wavelength of the therapeutic laser beam. Tumor irradiations were performed with four different laser wavelengths, i.e., 808 nm, 940 nm, 975 nm, and 1064 nm, using the same laser settings. Thermographic imaging was utilized for real-time temperature monitoring. Based on the measured temperature values, the quantitative thermal analysis was performed to assess the differences among breast cancer models treated with the diverse laser wavelength-gold nanorod combinations.

In this concern, the present work focuses on the evaluation of the thermal effects during *in vivo* photothermal treatments exerted at different radiation wavelengths and in combination with GNRs. The study plan is outlined in Figure 1. PTT was performed with different GNR sizes, each one associated with one out of four laser wavelengths, included in the therapeutic window. To investigate the thermal effects of GNR-assisted laser irradiation, experiments were performed on breast cancer tumors. The choice of this tumor model relies on the increasing request for the implementation of minimally invasive ablative techniques as surgical surrogates for the treatment of breast cancers characterized by reduced dimensions [29]. Indeed, contactless thermal ablation could represent an effective solution for the treatment of early-stage-diagnosed solid tumors affecting superficial organs, such as breast [30,31]. Therefore, in our study, subcutaneous breast tumors were grown on mice flanks, and thermographic imaging was used to assess the skin superficial temperatures reached during tumor irradiation. The thermal analysis investigated the differences among tumors directly injected with GNRs or phosphate-buffered saline (PBS), undergoing NIR laser exposure.

## Materials and methods

### Animal tumor model of breast cancer

All animal procedures were reviewed and approved by the City of Hope Institutional Animal Care & Use Committee and all experiments were conducted in accordance with relevant guidelines and regulations. Female, athymic nude mice characterized by 6–8 weeks of age (Charles River) were maintained under specific pathogen-free conditions at the City of Hope Animal Resource Centers. Before tumor cells inoculation, mice were anesthetized through 4% isoflurane in 1.5 L/min oxygen flow. Red firefly luciferase (Red-FLuc) 4T1 breast cancer cells were suspended in RPMI medium and Matrigel prior to injection. Then, mice were injected in the upper thigh with 10,000 4T1-Red-FLuc cells in 50 µL RPMI and 50 µL Matrigel. A digital caliper (± 0.01 mm) was used to measure tumor sizes, and tumor volume was calculated according to $V=L\text{ · }W^{2}/2,$ where V is the volume of the tumor in mm^3^, L (mm) is the longest dimension, i.e., tumor length, and W (mm) is measured perpendicular to L, i.e., tumor width. The calculated average pretreated tumor volume was 76.1 mm^3^.

### Assessment of viable engraftment

Sixteen days after tumor establishment, xenogen imaging was performed to confirm viable engraftment. A charge-coupled device camera (Xenogen IVIS-100, Xenogen Corporation, Almeda, CA) was used to image the firefly luciferase cancer cells. The nude mice were firstly anesthetized with isoflurane, then D-luciferin substrate suspended in PBS at 4.29 mg was administered per each mouse, while the whole procedure was performed in an induction chamber. The light emission was measured over an integration time of 10 s. A living image acquisition and analysis software (PerkinElmer, Inc., Waltham, MA) served for the resultant tumor flux analysis. Only after confirmation of viable engraftment by xenogen imaging, photothermal ablation was performed on breast cancer tumors.

### Administration of GNRs

GNRs were supplied by Nanopartz™, a division of Concurrent Analytical, Inc. The GNRs surface coating was selected to be 11-mercaptoundecyltrimethylammonnium bromide (MUTAB). MUTAB was preferred over other surface coatings, such as cetyltrimethylammonium bromide (CTAB), to avoid cytotoxicity issues due to the surfactant [20], since CTAB results toxic when dissociated from the GNRs [10,32]. Furthermore, MUTAB exploits peculiar characteristics thanks to the intrinsic capabilities to covalently bind to GNRs and favor the cellular uptake, due to its cationic charge [33], which makes this coating a valid candidate for biocompatible nanostructures. Four different laser wavelengths were used in the present study, i.e., 808 nm, 940 nm, 975 nm, and 1064 nm, therefore a specific GNR type was injected according to each specific wavelength. Thus, the aspect ratio of the nanostructure was selected in line with the wavelength of laser light to exploit the desired surface plasmon resonance (SPR phenomenon) [34]. The characteristics of the GNRs selected for each wavelength of the laser emitters are presented in Table 1. The GNR size, i.e., diameter (*D*) and length (*l*), the aspect ratio (AR) and the zeta potential (*ζ* potential) for GNRs dispersed in an 18 MOhm deionized water are displayed.

**Table 1.** Characteristics of the GNRs employed in this study.

D [nm]	l [nm]	AR	*ζ* Potential [mV]
10	41	4.1	41
10	55	5.5	41
10	58	5.8	39
10	67	6.7	39

The sizes of GNRs were chosen according to the laser wavelengths used to irradiate tumors.

**Figure 2.**
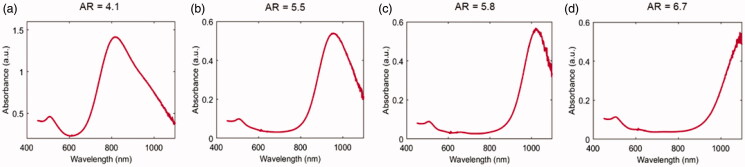
Vis-NIR absorbance spectra in a wavelength range of 450–1100 nm for the different GNR types. (a) AR = 4.1, (b) AR = 5.5, (c) AR = 5.8, (d) AR = 6.7.

The absorption spectra for all the types of GNRs, supplied by the manufacturer (Nanopartz™, Inc.), were attained in order to detect the GNRs absorption peaks and are shown in Figure 2.

Intratumoral administrations required anesthetizing the mice with isoflurane (4% isoflurane, 1.5 L/min oxygen flow), then a 28.5-gauge 0.5 cc syringe was used for injecting 20 µL of either GNR suspensions (12.5 µg) or PBS (1 X, pH = 7.4). The choice of using 12.5 µg of GNRs relies on the results of previous study in which different GNRs concentrations were compared for PTT purposes [35]. The injections were performed manually, and each injection took approximately 3 s.

### Laser irradiation

Three days post-intratumoral administration [20], contactless laser irradiation was performed. Four diode lasers (LuOcean Mini 4, Lumics, Berlin, Germany) with characteristic wavelengths of 808 nm, 940 nm, 975 nm, and 1064 nm, operating in a continuous-wave regime, were used. The laser power (P) was set at 2.6 W, with a beam spot size of ∼0.8 cm in diameter. A quartz optical fiber of 400 µm core diameter (numerical aperture of 0.22, OZ Optics Ltd., Ottawa, Canada) connected to a collimator delivered laser radiation. Tumors established in mice flanks were subdivided into eight treatment groups (*n* = 4/group), for a total of 32 subcutaneous tumors [20,36,37]. Four groups injected with GNRs underwent NIR-laser exposure according to: (a) GNRs (AR = 4.1)-injected tumors, laser irradiation at *λ* = 808 nm, (b) GNRs (AR = 5.5), *λ* = 940 nm, (c) GNRs (AR = 5.8), *λ* = 975 nm, (d) GNRs (AR = 6.7), *λ* = 1064 nm. The remaining four groups injected with PBS underwent laser irradiations at the same selected wavelengths (Figure 1).

To perform the photothermal treatment, mice were anesthetized with isoflurane (4% isoflurane, 1.5 L/min oxygen flow). The eyes of the mice were treated with optical lubricant ointment to protect them from drying. Subsequently, glycerin-swabbed tumors were irradiated (90 s exposure time).

The selected laser settings were defined according to the range of setting parameters often employed in photothermal procedures [27], [38–40]. Particularly, the 808 nm laser wavelength was used as reference wavelength for the choice of irradiation settings, since it represents one of the most widely utilized in GNR-mediated PTT [9,20,21,38,41].

### Real-time thermographic imaging

**Figure 3.**
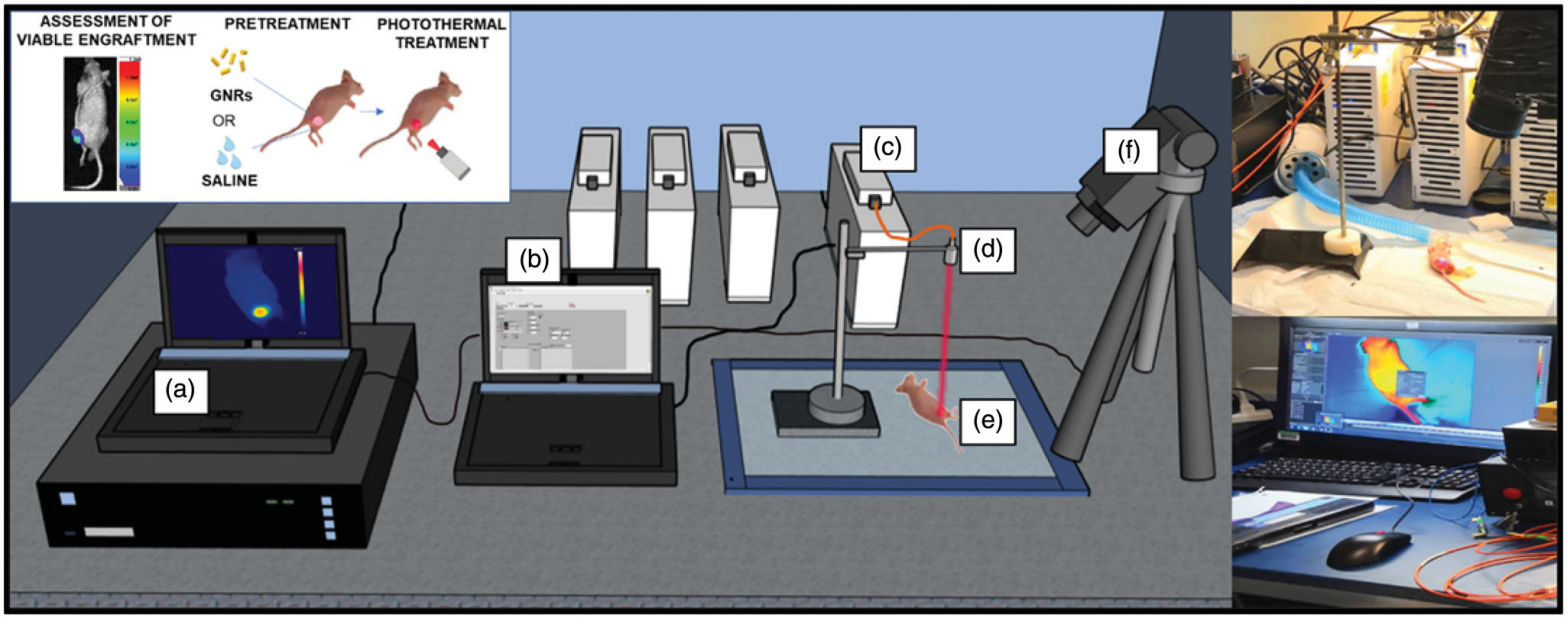
Experimental set-up to perform *in vivo* photothermal treatments on breast cancer tumors engrafted on mice flanks. It includes: (a) the computer for monitoring the temperature outcome, (b) the laser software for controlling the laser setting parameters, (c) the NIR laser emitter, (d) the collimator placed at applicator tip, (e) the anesthetized mouse bearing the breast cancer tumor, (f) the thermographic camera adopted for the measurement of the superficial temperatures.

An IR thermographic camera (FLIR System, A655sc, with 640 × 480 pixels spatial resolution, ±2 °C accuracy) was used to measure the surface temperatures of the treated tumor-bearing mice. Images were acquired at a frequency of 6 frames per second (f.p.s.). The experimental set-up is displayed in Figure 3.

### Data analysis

**Figure 4.**
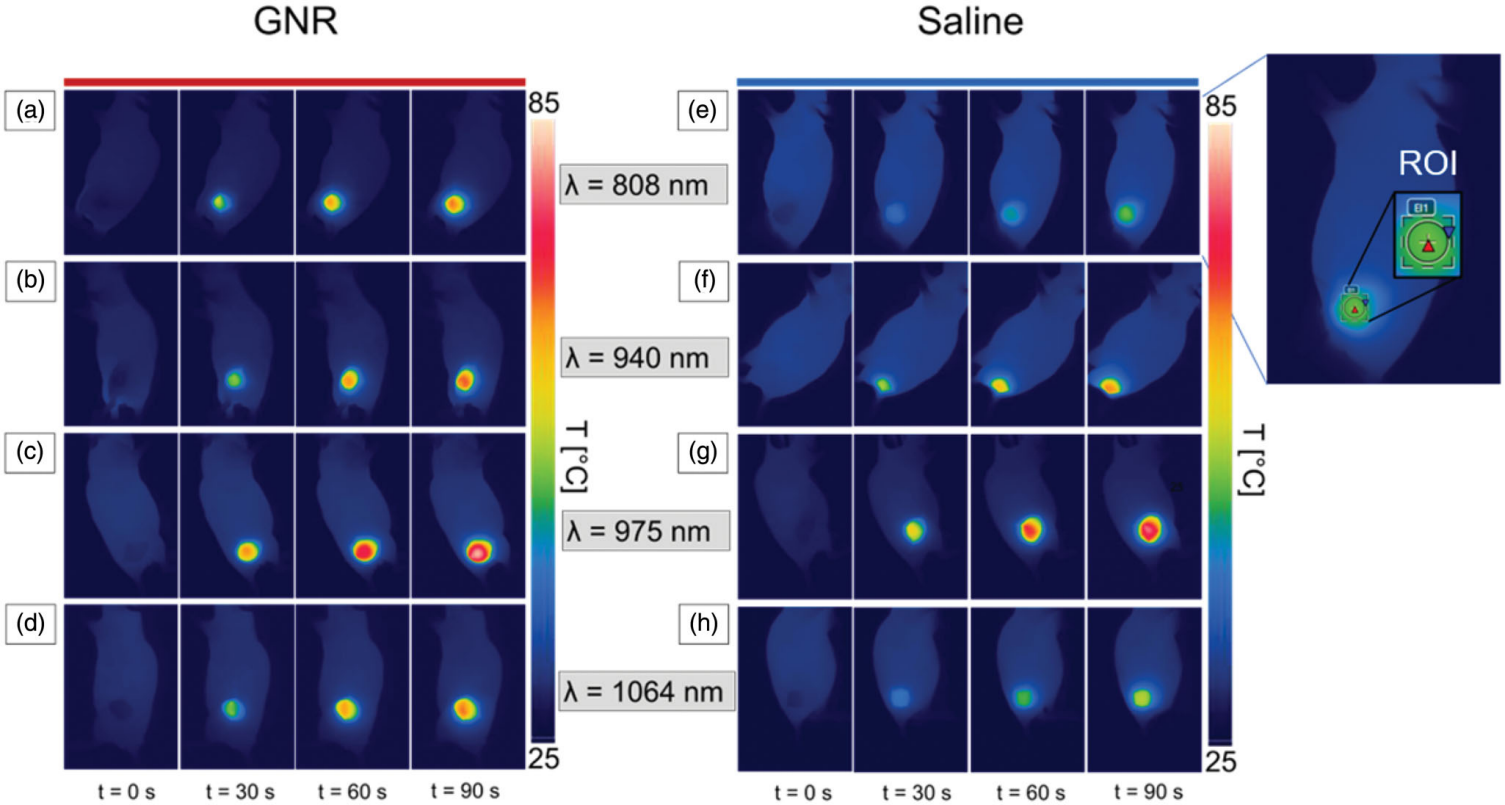
Thermographic images of subcutaneous 4T1 tumors at different exposure times of NIR laser irradiation, i.e., pretreatment (*t* = 0 s), 30 s, 60 s, and 90 s. Thermographs show the temperature values attained for GNR-treated tumors (a–d) and saline-injected tumors (e–h), irradiated at the different radiation wavelengths: 808 nm (a,e), 940 nm (b,f), 975 nm (c,g), and 1064 nm (d,h). An exemplificative image of the region of interest (ROI) defined in each thermograph is also reported.

Temperature measurements were collected for each tumor undergoing laser irradiation and registered throughout the whole procedure. In each thermal image, a region of interest (ROI) was defined as a circle of 30 pixel-diameter, located within the tumor area, encompassing the maximum temperature values (Figure 4). The mean temperature value and the standard deviation were calculated for each defined ROI. Measurement data were expressed as the mean ± standard deviation (SD). Differences in tumoral temperatures and thermal time constants between GNR- and saline-injected tumors were analyzed using the 2-tailed Student t-test. The differences between groups were considered statistically significant at a *p*-value lower than 0.05.

## Results

### *In vivo* photothermal treatment

The temperature values acquired with thermographic imaging for 808 nm laser irradiation indicated a mean value of the maximum temperatures ($T_{\text{max}}$) of 68 ± 2 °C and 52 ± 1 °C for GNRs-injected (12.5 μg/tumor) and saline control tumors, respectively, after 90 s of laser irradiation, in the selected ROI.

The heat distribution at the different exposure times in both GNR- and saline-injected tumors is shown in Figure 4. Here, the thermographs show the superficial temperature variation at different time intervals, i.e., *t* = 0 s, 30 s, 60 s and 90 s, for both tumors injected with GNRs (left) and PBS (right).

### Temperature results of NIR-irradiated tumors

**Figure 5.**
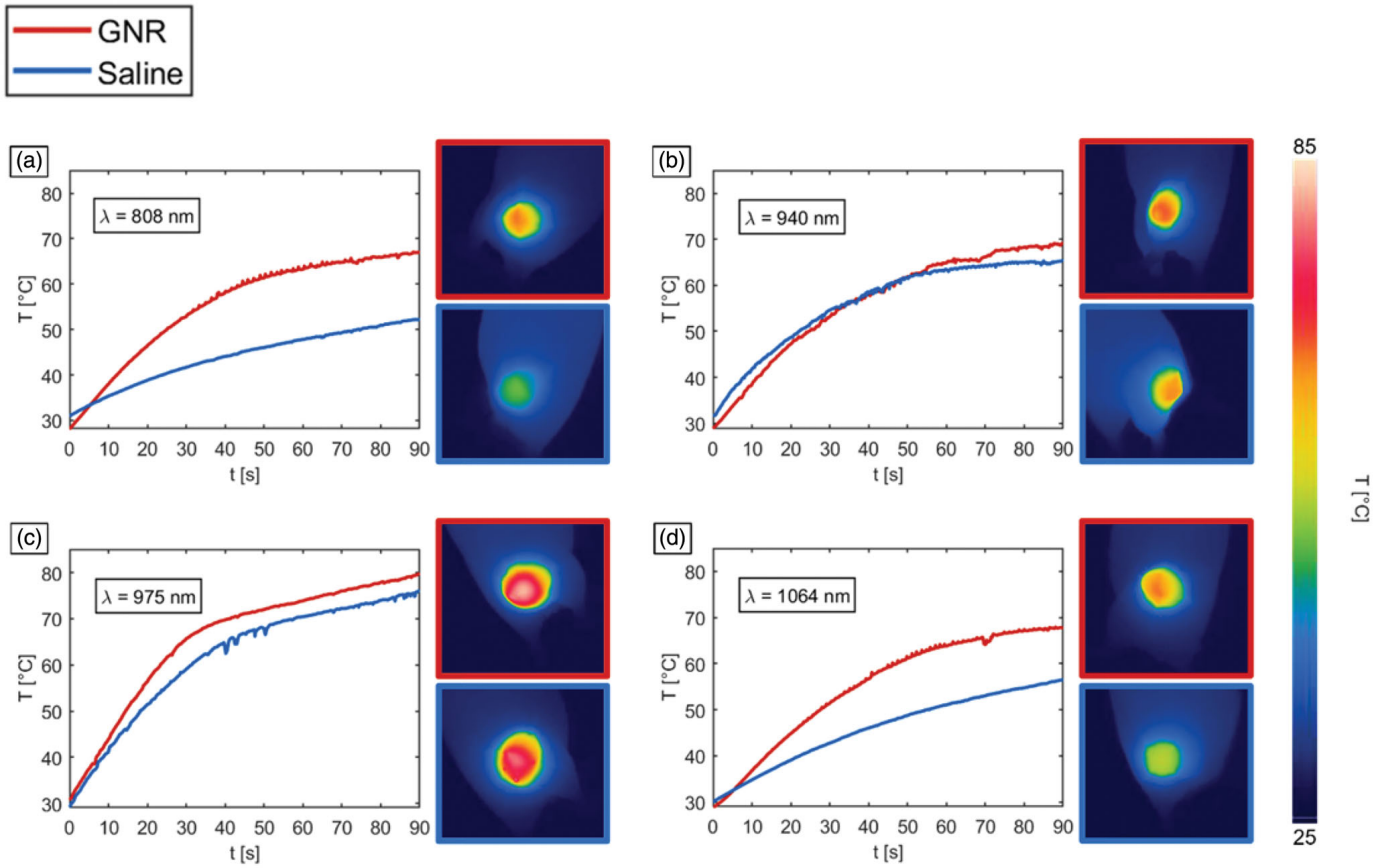
Temperature *vs.* time, and thermographs after a NIR-light exposure time of 90 s for GNR-injected tumors (red lines) and saline solution-loaded tumors (blue lines), undergoing laser irradiations at different wavelengths: (a) 808 nm, (b) 940 nm, (c) 975 nm and (d) 1064 nm.

In Figure 5, representative temperature trends registered for the irradiated breast cancer tumor models treated with the different GNRs and PBS are depicted for each radiation wavelength, together with the thermographs showing the superficial temperature distribution after 90 s-exposure time. The introduction of GNRs entailed an increased tumor temperature for all the differently irradiated groups. The fact that in Figure 5a,b, the temperature for GNRs group is lower than the temperature for saline group in a certain time interval can be ascribed to the higher initial temperature of the saline-injected tumor compared to the GNRs-loaded tissue (initial temperature difference of ∼3 °C).

**Figure 6.**
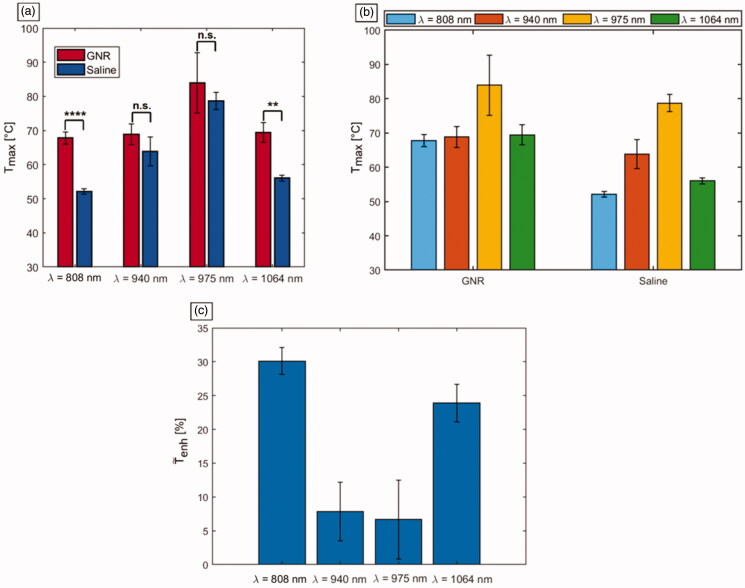
Mean values of the maximum temperatures ($T_{\text{max}}$) reached in tumors undergoing photothermal treatments. (a) Temperatures displayed according to the employed laser wavelengths. The significance is reported on the barplots: **** for *p* < 0.0001, ** for *p* < 0.01, non-significant (n.s.) difference otherwise. (b) Temperatures shown according to the injection groups, i.e., either GNRs with different ARs or saline solution. (c) Effective temperature enhancement (${\tilde{T}}_{\text{enh}}$) expressed in percentage, for the different GNR-laser wavelength combinations.

In Figure 6, the values of $T_{\text{max}}$ obtained for the ablated tumors are shown, for each employed laser wavelength (Figure 6a) and each treatment group (Figure 6b). Concerning the temperature reached in the control tumors, i.e., without GNRs assistance, the highest values were associated with 975 nm laser irradiation ($T_{\text{max}}$ = 79 ± 3 °C). Also, among all the tumors pretreated with GNRs, 975 nm laser was characterized by the highest temperature ($T_{\text{max}}$ = 84 ± 9 °C). The interaction of 940 nm laser light with control tumors displayed the temperatures closest to the ones reached by the 975 nm wavelength. However, the latter showed a $T_{\text{max}}$ of ∼15 °C higher than 940 nm radiation. The $T_{\text{max}}$ reached in GNRs-assisted PTT at *λ* = 940 nm was 69 ± 3 °C. The interaction of 808 nm and 1064 nm lasers with tumor tissue in PBS controls resulted in the lowest $T_{\text{max}},$ 52 ± 1 °C and 56 ± 1 °C, respectively. The temperature values were subject to an increase for both 808 nm and 1064 nm lasers when GNRs were injected. In this case, for 808 nm radiation, the reached $T_{\text{max}}$ was 68 ± 2 °C, whereas for the 1064 nm wavelength, a $T_{\text{max}}$ of 69 ± 3 °C was displayed. Therefore, for these radiation wavelengths, the temperature achieved in GNR-treated tumors was significantly greater than that in tumors injected with saline (*p* < 0.01). However, it remained approximately 10 °C lower than the superficial temperature exhibited by the 975 nm in laser-irradiated saline controls. Conversely, for 975 nm laser radiation, the temperature values achieved for GNRs-injected tumors were not significantly higher than in treated PBS controls (*p* = 0.324), with a temperature difference of ∼5 °C compared to PBS at the end of the laser discharge. A similar temperature increase compared to controls is also observable among GNRs-loaded tumors treated with NIR radiation at 940 nm. Thus, while in absence of nanoparticles the temperature differences for LA treatment at *λ* = 808 nm and 1064 nm were approximately 27 °C and 23 °C, respectively, compared to the values of 975 nm radiation (maximum temperature displayed in the treatment group type), the direct tumor administration of GNRs lowered this temperature difference. In fact, after the intratumoral injection of GNRs, the registered differences resulted in ∼16 °C between 975 nm and 808 nm procedures, and ∼14 °C concerning the treatments executed at *λ* = 975 nm and 1064 nm. Differently, regarding LA at 940 nm, the temperature differences with the values obtained for 975 nm remained constant at approximately 15 °C for both GNRs-administered and control tumors. In Table 2, the average temperature changes (Δ*T*) exhibited by the differently treated tumors are reported.

**Table 2.** Mean temperature change (Δ*T* [°C]) exhibited for GNR-injected tumors (GNR) and saline controls (Saline), undergoing laser irradiation at different laser wavelengths, and attained values of the heating efficiency (HE) associated to photothermal procedures.

Laser *λ* [nm]	Δ*T* [°C]		HE
	GNR	Saline	
808	39	22	1.8
940	40	33	1.2
975	55	50	1.2
1064	40	26	1.6

### Effective temperature enhancement and PTT heating efficiency

To investigate the effective temperature enhancement (${\tilde{T}}_{\text{enh}}$) exerted by GNR, the calculation of the maximum temperature difference between GNRs- and PBS-injected tumors, normalized to the maximum temperature in saline-injected tumors, was performed. Therefore, ${\tilde{T}}_{\text{enh}}$ was defined as reported in Equation (1):

(1) $${\tilde{T}}_{\text{enh}}\;=\text{ ((}T_{i}\;-\;T_{\text{PBS},i})/T_{\text{PBS},i}\text{) · }100$$

$T_{i}$ represents the mean maximum temperature achieved for the different i, i.e., GNR’s AR-laser wavelength combinations, and $T_{\text{PBS},i}$ is the mean maximum temperature reached with the same laser wavelength in the tumor injected with PBS (Figure 6c). The highest temperature enhancement was registered by 808 nm laser wavelength (${\tilde{T}}_{\text{enh}}$ ∼ 30%) due to the assistance of GNRs (AR = 4.1). Then, 1064 nm wavelength exhibited ${\tilde{T}}_{\text{enh}}$ of approximately 24% when tumors were injected with GNRs (AR = 6.7) compared to PBS. Then, 940 nm and 975 nm showed the lowest values, corresponding to ∼8% and ∼6%, respectively.

Furthermore, the heating efficiency (HE) of the photothermal procedure was obtained to evaluate the increased light to heat conversion allowed by GNRs, considering the temperature changes in both GNR-loaded and control tumors. HE was calculated for each wavelength as the ratio between the maximum temperature variation among the tumors injected with GNRs, undergoing the laser treatment, and the maximum temperature change among the NIR-irradiated controls [12,21]. The results were in accordance with the estimation of effective thermal enhancement. Indeed, the highest HE referred to the wavelength of 808 nm with a value of 1.8. Laser irradiation at 1064 nm showed a HE = 1.6, whereas 940 nm and 975 nm radiations were both characterized by a lower efficient thermal conversion of ∼1.2 (Table 2).

### Time constants analysis

**Figure 7.**
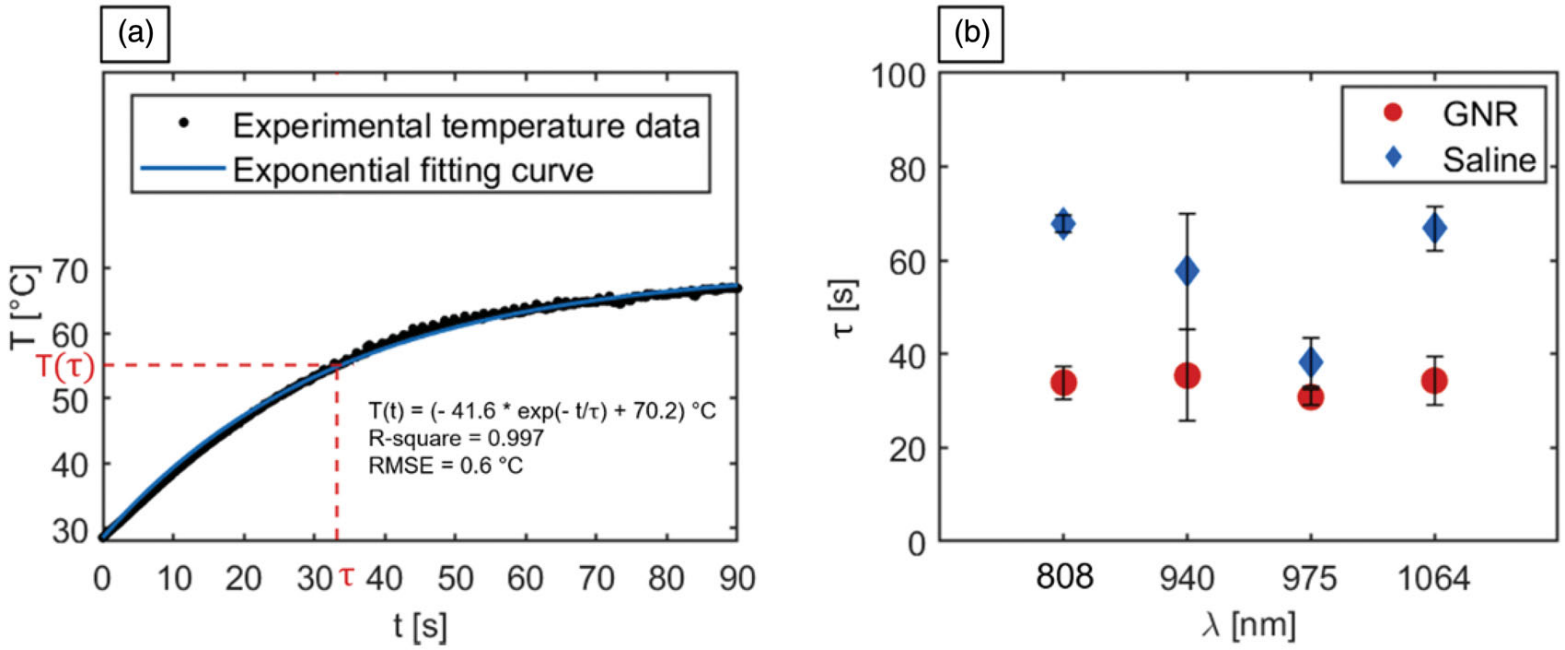
Analysis of the time constants associated with the temperature trends of laser irradiated tumors. (a) Representative graph depicting the experimental tumor temperature values measured during the photothermal treatment and the associated exponential fitting curve. The equation of the fitting curve, the R-square, and the RMSE are also reported. (b) Time constants (*τ*) associated with the temperature evolution of GNR-loaded and saline-injected tumors, undergoing laser exposure at the different selected wavelengths.

Referring to the step response of a first-order system as a model, the temperature graphs obtained through IR thermographic imaging were utilized to calculate the time constants associated with the irradiated tumors (Figure 7). The analysis was performed to attain a quantitative assessment of the laser-induced thermal response of the NIR-exposed biological tissues and investigate the implications of the introduction of highly light-absorbing nanoparticles on the tumor temperature evolution during PTT. The analysis of response times allows for the characterization of the system dynamics. This permits to investigate the time needed by the system (i.e., irradiated tumorous tissue) to reach a steady-state. The thermal response curve was expressed as reported in Equation (2):

(2) $$T(t)=(T_{0}\;-\;T_{\infty })\cdot \;{\text{exp}}^{\left(-\;\frac{t}{\tau }\right)}+T_{\infty }$$

T(t)  indicates the tumoral temperature at the instant of time *t*, $T_{0}$ is the initial temperature, $T_{\infty }$ represents the steady-state temperature value, and *τ* is the thermal time constant characterizing the system response. The values of *τ* were computed from the exponential fitting of the thermal curves of all the laser-irradiated tumors, with 95% confidence bound. The mean *R*-square was > 0.99, with a root mean square error (RMSE) ranging from 0.1 °C to 1.7 °C, with an average value of 0.7 °C. Figure 7a displays a representative graph of the experimental tumor temperature data acquired during the 90 s irradiation and the associated fitting curve. Figure 7b reports the *τ* values for each experimental setting. Among the tumors injected with saline solution, the 808 nm radiation was characterized by the highest thermal time constant, with a mean value of 67.8 ± 1.8 s. A comparable thermal response is shown by the tumors exposed to 1064 nm radiation (*τ* = 66.9 ± 4.8 s), while, concerning the treatment performed at 940 nm, a mean thermal time constant of 57.7 ± 12.3 s was obtained. The lowest mean *τ* among the PBS-treated tumors referred to 975 nm radiation (38.3 ± 5.1 s), which denotes the quickest laser-induced thermal response of the biological media among irradiated controls. Compared to control, the injection of GNR suspensions resulted in a decrease of the thermal time constants for all the wavelengths. Particularly, the tumors irradiated at 808 nm and 1064 nm experienced a substantial reduction of ∼33 s in *τ*, with respect to the values attained in LA of saline-injected tumor models. The group of tumors exposed to 940 nm laser light also showed a lowered mean value of the thermal time constant in case of GNR assistance (35.4 ± 9.8 s) compared to saline-injected tumors. As observed in laser-treated controls, the characteristic wavelength radiation of 975 nm was associated with the minimum value of *τ* also for GNR-mediated PTT. However, at this radiation wavelength, the difference in terms of tissue thermal response in GNR-loaded irradiated tumors resulted only ∼7 s lower than PBS-injected tumors.

**Figure 8.**
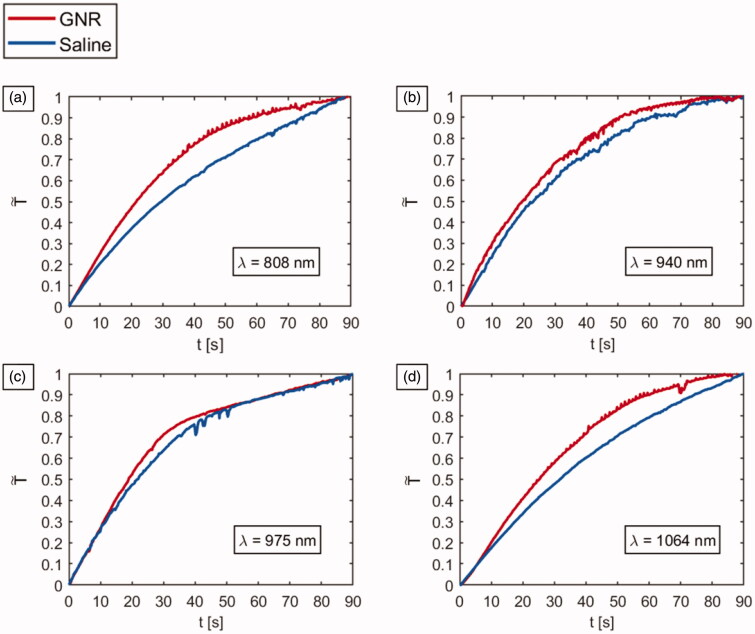
Trends of $\tilde{T}$ vs. time for tumor injected either with GNRs (red line) or saline (blue line) subject to NIR-laser irradiation at the different radiation wavelength: (a) 808 nm, (b) 940 nm, (c) 975 nm, and (d) 1064 nm.

The first-order characteristics of the tumors with and without GNRs are compared in Figure 8. The values of $\tilde{T},$ defined as temperature change at each instant of time normalized to the maximum temperature change, over time, are reported to better characterize the heating kinetics of the differently treated tumors. $\tilde{T}$ was calculated according to Equation (3):

(3) $$\tilde{T}\;=\;(T(t)\;-\;T_{\text{min}})/(T_{\text{max}}\;-\;T_{\text{min}})$$

T(t) is the temperature measured at the instant of time *t*, $T_{\text{min}}$ refers to the minimum tumor temperature, and $T_{\text{max}}\;$ is the maximum temperature experienced by the tumor exposed to laser radiation. In the case of irradiations performed at 940 nm and 975 nm, similar trends for GNR-loaded tumors and saline-injected controls are observable. The thermal behavior of 975 nm-irradiated tumors results particularly noteworthy since similar values of $\tilde{T}$ are observable for GNR- and PBS-treated tumors for almost all the irradiation time. Concerning the tumors exposed to 808 nm and 1064 nm NIR-laser light, the differences in heating kinetics between GNR and saline groups result more remarkable than in the previous cases, displaying a clear change of the trend of $\tilde{T}$ over time, subsequent to the nanoparticle injections.

## Discussion and conclusions

In this study, a quantitative thermal analysis of LA assisted by GNRs at different laser wavelengths of the therapeutic window is devised, to investigate the thermal behavior of irradiated tissue, in view of PTT applications. Indeed, the introduction of GNRs in the cancer region and the subsequent irradiation at optimized laser settings may lead to a cytotoxic temperature increase only in the desired area, hence enhancing the procedure selectivity. However, the optimized conditions for the safe delivery of an efficient thermal dose, are still an open question.

Here, different GNR aspect ratios, each one tailored with one out of four laser wavelengths, i.e., 808 nm, 940 nm, 975 nm, and 1064 nm, to show the SPR peak at the wavelength of stimulation, were used. *In vivo* GNR-mediated PTT was executed on 4T1-breast cancer tumors, grown on mice flanks. The same laser settings, chosen according to the range of setting parameters typical of PTT [13,27,38,40], were maintained for all the treatments to obtain confrontable results. At the specified settings, in presence of GNRs, the 808 nm irradiation showed tumor maximum temperatures which lay in the thermal range known to induce therapeutic effect (58–70 °C) [42], due to substantial protein denaturation and necrosis within seconds [1,42–44]. Whereas, in controls, the light-to-heat conversion exerted by endogenous chromophores resulted in lower temperatures (∼52 °C), which should not induce irreversible damage if maintained for reduced periods. The effective temperature enhancement due to the injections of nanoparticles resulted in the highest one among the considered laser light-GNR combinations (30%). Moreover, also the heating efficiency of PTT showed the highest value among all the groups, approximately 1.8, which is consistent with the results attained in previous studies for direct tumor injection of GNRs in human breast cancer xenografts (1.69) [21], and intravenous administration of pegylated GNRs in squamous cell carcinoma in mice (∼1.9) [12]. The injection of GNRs determined a variation in terms of temperature evolution in time, denoted by a reduced thermal time constant compared to laser-irradiated controls. Therefore, the presence of GNRs resulted in a quicker laser-induced thermal response of the tumor samples. The observed temperature trend may refer to the overall increase of the absorption coefficient of the irradiated media [45], thus to the corresponding enhanced photothermal conversion capability due to GNRs injection. Considering a specific wavelength, once GNRs are injected, the conversion of absorbed light into heat is regulated by the combination of the optical properties of the nanostructures and the biological chromophores, active at that light wavelength. The heating kinetics of the groups of tumors injected with GNRs present similar values of time constants (maximum difference of around 5 s). This suggests that the introduction of highly light-absorbing photosensitizer, with SPR tuned according to the laser wavelength, may change the overall nanoparticle-loaded tissue optical behavior, thus leading to a faster optothermal response, regardless of the adopted wavelength, compared to controls. As a matter of fact, in case of irradiation of biological tissue without GNR, the thermal response is dominated by the intrinsic optical characteristics of the wavelength-dependent endogenous structures. Thus, in this case, major differences in time constants among the diverse laser wavelengths can be observed.

Tumor irradiation performed at the wavelength of 1064 nm yielded an overall thermal behavior, in terms of maximum temperatures and thermal time constant, similar to the previous one described for 808 nm radiation procedures. Indeed, the temperature in GNR-mediated photothermal treatments (>58 °C) could trigger cell death-related phenomena. Whereas, in PBS-injected tumors, temperatures were maintained below 60 °C. However, slightly higher values of temperature in controls and a lower PTT heating efficacy (1.6) are associated with 1064 nm radiation, when compared to 808 nm. The presented difference in results could be ascribed to the optical characteristics of the breast cancer model, i.e., an animal stage IV human breast cancer, at these specific wavelengths. For instance, our results are in concordance with the absorption spectra obtained from *in vivo* human breast tissue measurements which showed an increased absorption coefficient at 1064 nm, compared to 808 nm [46,47]. Therefore, higher temperatures in controls for 1064 nm laser compared to 808 nm-irradiated tumors can be explained in terms of intrinsic tissue optical properties. The effective temperature enhancement, which refers to the temperature arisen due to the capability of GNRs as photothermal traducers compared to the temperature in controls, resulted lower for 1064 nm irradiation (24%) than for 808 nm. However, at both these radiation wavelengths, and employed settings, irradiated tumors underwent a substantial temperature increase when loaded with GNR photosensitizers compared to PBS, thus presenting promising results in terms of improvement of the procedure selectivity.

Breast cancer tumor models treated with the 940 nm-laser exhibited a temperature increase which may cause irreversible thermal damage [42], even among controls (64 °C). Conversely, the tumor group injected with GNRs showed temperatures only approximately 5 °C higher than controls, with almost four-fold lower effective temperature enhancement than in the case of 808 nm laser treatment. The obtained thermal results displayed when 940 nm interacted with engrafted tumors are in line with the absorption properties of biological tissues [46,48]. In fact, close to the emission peak of the employed stimulating laser radiation, the absorption peak of lipids lies at 930 nm [46]. Hence, increased absorption and a reduced penetration depth into biological structures [22] may explain the attained higher superficial temperatures compared to 808 nm and 1064 nm irradiations, in controls. Moreover, our results are in accordance with a previous investigation concerning the comparison of 940 nm and 1064 nm radiations during interstitial laser-induced thermotherapy, which reported the creation of coagulation volume after short times with 940 nm irradiation, due to lower optical penetration depth [22]. Concerning the treatments of tumors irradiated at a laser wavelength of 975 nm, the attained temperature values were >70 °C, in both PBS controls and GNR-injected tumors, resulting in the highest temperature increase displayed among all the radiation wavelengths. These thermal values typically refer to tissue modifications, which can reduce the diffusion of heat through the deeper cancer tissue [43]. The temperature results are consistent with the absorption characteristics of biological tissues since the adopted stimulating radiation corresponds to the absorption peak of water, i.e., 975 nm [46,49]. Hence, the augmented absorption coefficient can explain the high photothermal heat conversion and superficial temperature rise compared to the other radiation wavelengths, in controls. The effective temperature enhancement due to GNR injections was lower than in the other GNR-laser wavelength combinations, with a low PTT heating efficiency, similar to the one shown during irradiation at 940 nm. The temperature evolution associated with 975 nm laser resulted in the quickest thermal response of biological tissue, corresponding to the lowest time constants in both GNR- and saline-injected tumors. A result that is in line with the outcome of a preclinical assessment of LA executed on large animal model, which showed large ellipsoid thermal ablations in less than 3 min [23]. However, in GNRs-treated tumors, the mean time constant associated with the thermal response was not significantly lower than in controls. A possible explanation of the observed reduced influence of GNR injections in terms of effective temperature enhancement and decrease of time constant (i.e., thermal response of NIR-treated tumors) for 975 nm and 940 nm irradiations may refer to the reported high light absorption exerted by endogenous chromophores, at these wavelengths and the correlated low optical penetration depth, compared to 808 nm and 1064 nm. Indeed, according to previous studies which investigated the GNR biodistribution, the intratumoral injections of GNRs in triple-negative breast cancer xenografts confined the GNRs at the center core of the tumor, with a smaller quantity located at the periphery [20,35]. Therefore, a high tissue light-absorption at the tumor surface may impede the laser light penetration to the inner part of the cancer lesion, thus the interaction with the highest concentration of injected GNRs.

Considering the abovementioned aspects, the displayed thermal outcomes stand as a preliminary validation of the diffuse use, in literature, of 808 nm laser radiation in nanostructure-enhanced PTT [9,20,21,50] and the usage of 1064 nm laser in LA treatment of solid tumors [51]. Furthermore, they suggest that laser wavelengths of 808 nm and 1064 nm could be exploited with a proper dose of GNRs during nanoparticle-assisted PTT when an enhanced treatment selectivity is necessary and during thermal therapies when an increased light penetration depth is required. The possibility to optimally select the laser settings to ensure a harmless temperature increase in healthy tissue and to localize the cytotoxic thermal effect to the circumscribed tumoral region, in which the nanoparticles are located, may hold great promise for the usage of 808 nm and 1064 nm lasers in PTT, thanks to the related reduced absorption coefficients of tissue at these wavelengths compared to other wavelengths of the NIR window.

Moreover, our results show that also the 940 nm and 975 nm laser radiations may be employed, at accurately selected laser settings, for more elevated heating kinetics in tissue. Indeed, 940 nm and 975 nm wavelengths could be beneficial during interstitial therapies requiring lower optical penetration of light in tissue, i.e., when it is possible to directly reach the target organ by means of the optical fiber and control the laser light to attain the desired tumor necrosis. In these cases, a reduced optical penetration depth of the therapeutic laser beam, compared to 808 nm and 1064 nm, might be preferred to reduce the thermal dose delivered to healthy tissue located at deeper sites with respect to the tumor region [52]. Therefore, in these applications, 940 nm and 975 nm radiations could be utilized, without the necessity to administer nanoparticles, by accurately tuning the laser settings according to the specific tumor characteristics. Particularly, the 975 nm laser demonstrated the capability of producing rapid heating of tissues, as assessed by the lowest value of thermal time constants concerning irradiated tumors engrafted on mice. In accordance with our findings, studies have evaluated the use of these wavelengths for interstitial laser ablation (at 980 nm laser wavelength) [53,54] and the attainment of coagulation volume after short times (at 940 nm radiation) [22].

Starting from our results, future studies should investigate the optimization of the laser settings parameters to guarantee safe treatment margins [55,56], through the assessment also of the internal tumor temperature change during irradiation. Concerning the temperature monitoring approach utilized in the present study, thermographic imaging has been exploited thanks to its noninvasive nature and the possibility of accurate and real-time monitoring, which make it an excellent solution for the evaluation of the attained superficial temperature. However, with such a technique the internal temperature change is not possible to assess. Thus, other temperature monitoring methods should be employed to evaluate the temperature in the tumor volume. For instance, it could be assessed by the use of thermocouples and minimally-invasive fiber optic sensors, which have already shown promising results for thermal monitoring during PTT of breast tumor models [20,21]. Additionally, magnetic resonance imaging has been presented to enable the reconstruction of two- and three-dimensional thermal maps to attain an estimation of temperature change of the cancerous region and the surrounding healthy tissue during PTT [57].

Moreover, the implementation of imaging techniques [58] and the use of cells as delivery vectors could be exploited for increasing the uniform nanoparticle distribution inside tumors for inducing an all tumor-covering temperature increase [35,59].

In conclusion, this study extensively investigates and compares the thermal effect of different GNR-laser wavelength combinations during laser-assisted photothermal treatments of breast cancer tumors. Our quantitative thermal analysis, based on the diverse tissue responses and thermal kinetics attainable under different wavelengths of the therapeutic window and in combination with GNR photosensitizers, may provide further insight into the laser-tissue interaction and subsequent thermal behavior of irradiated tumors models encouraging future studies toward the minimally invasive treatment of focal malignancies affecting breast tissue.

Indeed, in PTT, the evaluation of the temperature evolution is of paramount importance as the prime factor triggering the desired damage to tumorous tissue. However, for the definition of the optimal ablation strategy, future investigations should also focus on the correlation of the temperature results attained at different wavelengths of laser irradiation and GNR combinations with the biological response and final thermal ablation outcome.
